# Superpulsed **CO**_**2**_ Laser with Intraoperative Pathologic Assessment for Treatment of Periorbital Basal Cell Carcinoma Involving Eyelash Line

**DOI:** 10.1155/2014/931657

**Published:** 2014-10-13

**Authors:** Ali Ebrahimi, Mansour Rezaei, Reza Kavoussi, Mojtaba Eidizadeh, Seyed Hamid Madani, Hossein Kavoussi

**Affiliations:** ^1^Kermanshah University of Medical Sciences (KUMS), Kermanshah, Iran; ^2^Health School, Family Health Research Center of Kermanshah University of Medical Sciences (KUMS), Kermanshah, Iran; ^3^Hajdaie Dermatology Clinic, Golestan Ave, Kermanshah 6714653113, Iran

## Abstract

*Background*. Periorbital basal cell carcinoma (BCC) is considered a high risk case because it is associated with high rate of recurrence and complication. Superpulsed CO_2_ laser with intraoperative pathologic assessment could be an alternative and appropriate treatment for periocular lesions where Mohs micrographic surgery is not available. *Objective*. To evaluate the efficacy of superpulsed CO_2_ laser therapy with intraoperative pathologic assessment on periocular BCC involving eyelash line. *Method*. This follow-up study was performed on 20 patients with a total of 21 BCC lesions that were pathologically documented. Firstly, debulkation of tumoral mass was done by curettage. Then, irradiation and intraoperative pathologic evaluation were done by concurrent CO_2_ laser. The patients were followed up for a period of 36 months. *Results*. Out of 21 lesions, the nodular type accounted for 15 (71.4%) lesions, and 12 (57.1%) lesions were seen in the lower lid as the most common clinical type and site involvement. Twenty BCC lesions (95.2%) were treated after one session. Damage to eyelash was seen in 2 (10%) patients, but ectropion and other complications were not seen in any patient. *Conclusion*. Treatment with superpulsed CO_2_ laser and intraoperative pathologic evaluation for periorbital BCC lesions much close to conjunctiva could be an effective method with minimal complications without major danger of recurrence. This modality can be used with care in the inner canthus and high risk pathologic lesions.

## 1. Introduction

Although basal cell carcinoma (BCC) is the most common malignant tumor of periorbital area, it rarely results in death [1–3].

BCC is associated with disfiguration and very high cost especially in large lesion, recurrent forms, aggressive pathologic subtype, poorly defined tumor, immunosuppression, and high risk locations such as periorbital region [1, 4, 5].

The periorbital BCC is the most common cause of orbital exenteration, especially in recurrent BCCs, infiltrative pathologic subtype, and medial canthal lesions [6].

Several optional treatments have been suggested for periorbital BCC such as chemotherapy [7, 8], traditional surgical excision [9–11], photodynamic therapy [12, 13], Mohs micrographic surgery [14–16], and laser ablation [17–20].

The use of superpulsed mode of CO_2_ laser compared with its traditional one results in precise destruction of lesion with minimum damage to the normal surrounding tissue due to minimal thermal diffusion; therefore, it is associated with low risk of hypertrophic or atrophic scar [21].

This study was carried out to evaluate the treatment outcome and complications of the superpulsed mode CO_2_ laser with concomitant pathologic assessment of periorbital BCC treatment.

## 2. Methods

This clinical follow-up study was carried out on 20 patients at Hajdaie Dermatology Clinic of Kermanshah University of Medical Sciences in Iran over a period of 48 months from 2007 to 2012. Biopsy was done in the patients that were clinically suspected of periorbital BCC extended to eyelash line. The patients with histopathologically documented BCC were enrolled in our study. Patients were given information about this procedure and asked for their consent. We consulted with ophthalmologist about any ocular problems and existence of any contraindication in the patients. The exclusion criteria included lesions with a diameter larger than 2 cm, pregnancy, patients younger than 30 years old, recurrence after excision, wide extension to conjunctiva, morphoeic form, immunosuppression, keloid former, and any orbital contraindication for laser therapy.

**Figure 1:**
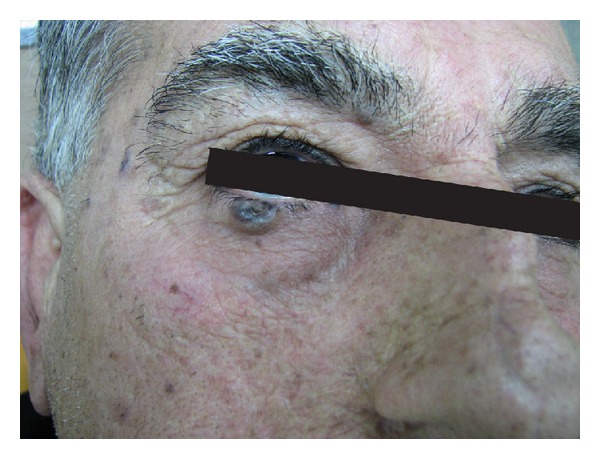
A man with 2 BCC lesions in lower lid.

**Figure 2:**
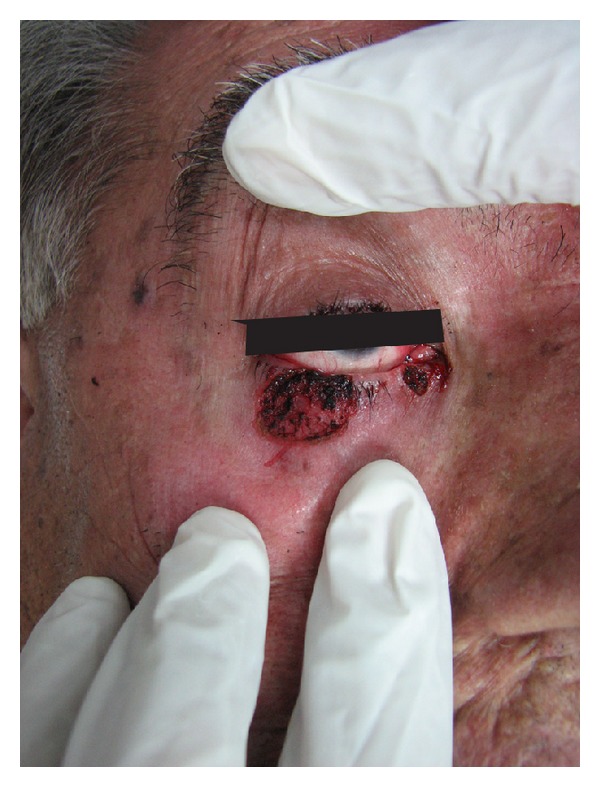
Induced defects after laser therapy.

We delineated 3 mm of normal appearing marginal skin around the BCC and this region was anesthetized with an injection of lidocaine 2% with or without epinephrine 1/100000, if there was no contraindication of epinephrine. The tumoral mass of BCC was removed by a very sharp curettage that resulted in an even defect. We treated the induced defect and marginal skin by 4 passes of superpulsed CO_2_ laser with appropriate eye protection. We selected the following laser therapy parameters (12-watt power and 600–800-microsecond pulse duration), and between laser passes the char was wiped away with saline-soaked gauze (Figures 1 and 2).

**Figure 3:**
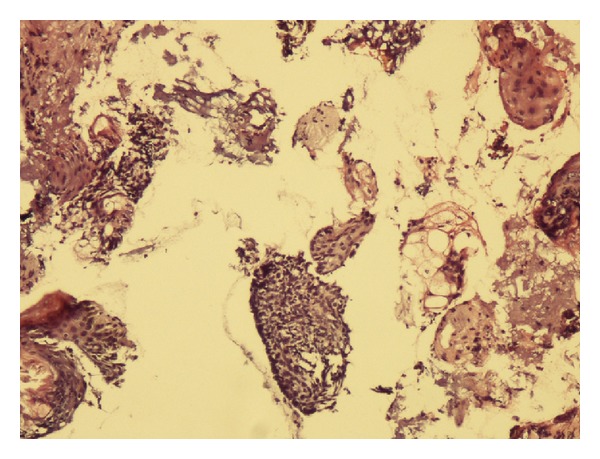
Shave sample of induced defect after CO_2_ laser indicates presence of malignant cells (H&E stain ×100).

In the end of procedure, the histopathological sample was obtained by a very sharp curettage from the base and margin of the treated site. In the presence of any malignant cells (Figure 3), retreatment was done by CO_2_ laser. This cycle of laser therapy and histopathology evaluation was performed until no malignant cells were seen.

**Figure 4:**
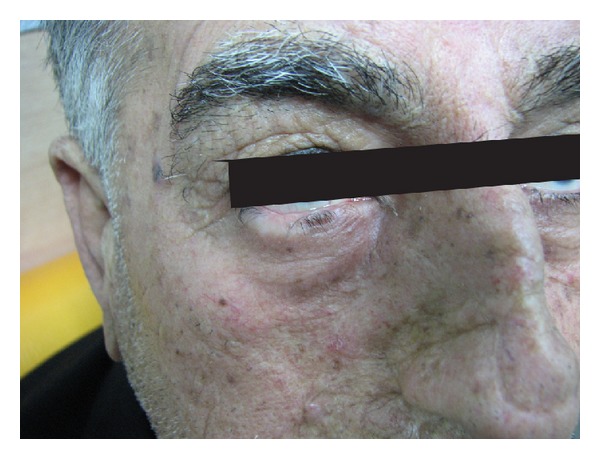
Six months after laser treatment.

Postoperative care included washing with normal saline and dressing with tetracycline ophthalmic ointment for 7–10 days. The induced defect was repaired by secondary intention (Figure 4).

This study was approved by the Ethics Committee of Kermanshah University of Medical Sciences and registered in the IRCT database (IRCT201404036403N4).

Analysis of data was carried out using the SPSS software version 16. Analysis of qualitative data was done by Chi-square and Fisher's exact test, and KS test was used for analysis of quantitative data. Levene's and the independent sample *t*-test were also used for comparison of variance and the means.

## 3. Results

Our study recruited 20 patients (7 females and 13 males) with 21 lesions. The age range of participants was between 42 and 80 with mean age of 61.43. The mean size of lesions was 10.62 mm (ranged between 5 and 20 mm) (Table 1).

The lesions were located in lower lid, inner canthus, upper lid, and outer canthus 12 (57.1%), 7 (33.3%), 1 (4.8%), and 1 (4.8%), respectively (Table 2).

The most common clinical and histopathological forms were nodular and solid.

The cure rate was observed in 20 (95.2%) lesions and recurrent rate was seen in 1 (4.8%) lesion in the follow-up period (Table 2).

Because there was 1 recurrence, it was not possible to run statistical test between recurrence and other variables.

Recurrence was seen in a 75-year-old male patient with nodular clinical lesion and infiltrative pathology with 20 mm diameter at inner canthus.

Damage to eyelash was seen in 2 (10%) cases, but other complications such as ectropion, trichiasis, atrophic and hypertrophic scar, and damage to eye structure were not seen in any patient (Table 2).

## 4. Discussion

Superpulsed CO_2_ laser with intraoperative histopathological evaluation is a highly appropriate modality for the treatment of periorbital BCC with high cure rate (95.2%) and low complication rate during 36 months of follow-up period.

The aim of periorbital BCC treatment is eradication of the tumor to prevent local recurrence, good aesthetic outcome, and preservation of lid function without any injury to eye structure [9].

The best treatment for BCC is Mohs micrographic surgery, a method of tumor removal with histologic margin control for residual malignant cells, which is superior to other treatments. However, it is expensive and time consuming and requires skilled surgical and pathological team [14–16]; it is also not generally available in most areas of the world including Iran.

Determination of BCC pathologic subtype in order to appropriate treatment is very important [22].

High recurrence rate of BCC in eyelid area must be expected according to histopathological type [23].

Cystic and nodular histopathologic subtypes of BCC are relatively well defined margin, but morphoeic, micronodular, infiltrative, and basosquamous BCCs have frequently ill-defined margin and are considered as high risk or aggressive histopathologic subtypes [5].

Traditional and new versions of CO_2_ laser were used for treatment of BCC on the head and neck and other sites of body [17–21, 24–29], but Bandieramonte et al. [17] reported the use of CO_2_ laser microsurgery in the treatment of 26 superficial BCC tumors combined with intraoperatory histopathological examination. They concluded that CO_2_ laser microsurgery appears to be the most effective treatment method only for primary superficial BCC of the eyelid margins without any complication.

Humphreys et al. [26] used pulsed CO_2_ laser for the treatment of primary superficial BCC and concluded that ultrapulse CO_2_ laser is the most favorable treatment for superficial BCC.

Campolmi et al. [27] treated 140 patients with superficial and nodular BCC by superpulsed CO_2_ laser. In the end of laser therapy, the bed of the treated site was excised for histopathological examination. This technique, in addition to clinical efficacy for superficial BCC, is associated with minimal thermal damage to the surrounding tissue and permits intraoperative histopathological evaluation.

Multiple passes of pulsed mode CO_2_ laser combined with intraoperatory histopathological examination have been used for the treatment of 21 superficial, 28 nodular, and 2 infiltrative BCC tumors, but laser ablation is a reliable method for patients with multiple superficial BCCs [28].

Previous studies have treated special clinical and histopathological types of BCC mostly on the trunk by CO_2_ laser, but we treated various clinical (nodular, superficial, and pigmented) and histopathological types of BCC on the periorbital area that involved eyelash line by superpulsed CO_2_ laser.

We performed concurrent histopathological study for complete removal of malignant cells and prevention of local recurrence as well as preservation of marginal normal tissue and consequently prevention of complications such as ectropion.

The anatomic distortion and scar induced following incomplete excision and repair of primary BCC obscure the malignant cells, which leads to recurrence and identification of tumor margin becomes more difficult [30, 31].

One of the main advantages of this form of therapy in contrast to surgical excision is that it induces no anatomic distortion. Therefore, any remaining malignant cells during laser therapy do not result in irregular growth of malignant cells; it even results in easy and early detection and extent of tumor [20].

BCC on the periorbital area not only is considered a high risk tumor [1–5] but also is associated with a number of complications such as ectropion, trichiasis, and damage to eyelash after surgical excision [32, 33].

In our method, the recurrence rate was reported in 1 (4.8%) lesion, which occurred in a BCC on the medial canthal lesion, infiltrative histopathological subtype with 20 mm diameter.

Damage to eyelash was seen in 2 (10%) patients, one in the lower lid with infiltrative pathologic subtype and another in medial canthal BCC, both of which had a diameter more than 10 mm. Therefore, patients need to be informed about the probability of eyelash damage in periorbital BCC with high risk infiltrative pathologic subtype and diameter more than 10 mm.

## 5. Conclusion

Our study indicated recurrence occurring in one case of nodular clinical type with 20 mm diameter and infiltrative histopathological subtype in the medial canthal lesion. Therefore, this method is an appropriate modality for small, other than inner canthal region, and nonhigh risk histopathological subtype but should be used with caution for large and high risk histopathological subtype in the medial canthal region.

## Figures and Tables

**Table 1:** Characteristics of patients.

Variables	Number
Number of patients	20
Sex of patients	
Female	7
Male	13
Number of lesions	21
Mean of age	61.43
Mean of size	10.62

**Table 2:** Characteristics of lesion, outcome, and complication of treatment.

Variables	Frequency	Percent
Clinical type		
Nodular	15	71.4
Pigmented	5	23.8
Superficial	1	4.8
Histopathologic subtype		
Solid	15	71.4%
Cystic	2	9.5%
Superficial	1	4.8%
Infiltrative	1	4.8%
Micronodular	1	4.8%
Basosquamous	1	4.8%
Location of treatment		
Lower lids	12	57.1
Medial canthal	7	33.3
Lateral canthal	1	4.8
Upper lids	1	4.8
Outcome		
Cure	20	95.2
Recurrence	1	4.8
Complication		
Damage to eyelash	2	9.5
Atrophic and hypertrophic scars	0	0
Ectropion	0	0
